# Transforming Ti_3_C_2_T_x_ MXene’s intrinsic hydrophilicity into superhydrophobicity for efficient photothermal membrane desalination

**DOI:** 10.1038/s41467-022-31028-6

**Published:** 2022-06-08

**Authors:** Baoping Zhang, Pak Wai Wong, Jiaxin Guo, Yongsen Zhou, Yang Wang, Jiawei Sun, Mengnan Jiang, Zuankai Wang, Alicia Kyoungjin An

**Affiliations:** 1 School of Energy and Environment, City University of Hong Kong, Tat Chee Avenue Kowloon, Hong Kong, Hong Kong; 2 Department of Mechanical Engineering, City University of Hong Kong, Tat Chee Avenue Kowloon, Hong Kong, Hong Kong

**Keywords:** Solar thermal energy, Two-dimensional materials

## Abstract

Owing to its 100% theoretical salt rejection capability, membrane distillation (MD) has emerged as a promising seawater desalination approach to address freshwater scarcity. Ideal MD requires high vapor permeate flux established by cross-membrane temperature gradient (∆T) and excellent membrane durability. However, it’s difficult to maintain constant ∆T owing to inherent heat loss at feedwater side resulting from continuous water-to-vapor transition and prevent wetting transition-induced membrane fouling and scaling. Here, we develop a Ti_3_C_2_T_x_ MXene-engineered membrane that imparts efficient localized photothermal effect and strong water-repellency, achieving significant boost in freshwater production rate and stability. In addition to photothermal effect that circumvents heat loss, high electrically conductive Ti_3_C_2_T_x_ MXene also allows for self-assembly of uniform hierarchical polymeric nanospheres on its surface via electrostatic spraying, transforming intrinsic hydrophilicity into superhydrophobicity. This interfacial engineering renders energy-efficient and hypersaline-stable photothermal membrane distillation with a high water production rate under one sun irradiation.

## Abstract (web summary)

Membrane distillation is susceptible to thermal inefficiency and membrane wetting issues during seawater desalination. Here, authors design a MXene-engineered membrane that imparts efficient localized photothermal effect and strong water repellency, achieving sustainable freshwater production.

## Introduction

**Fig. 1:**
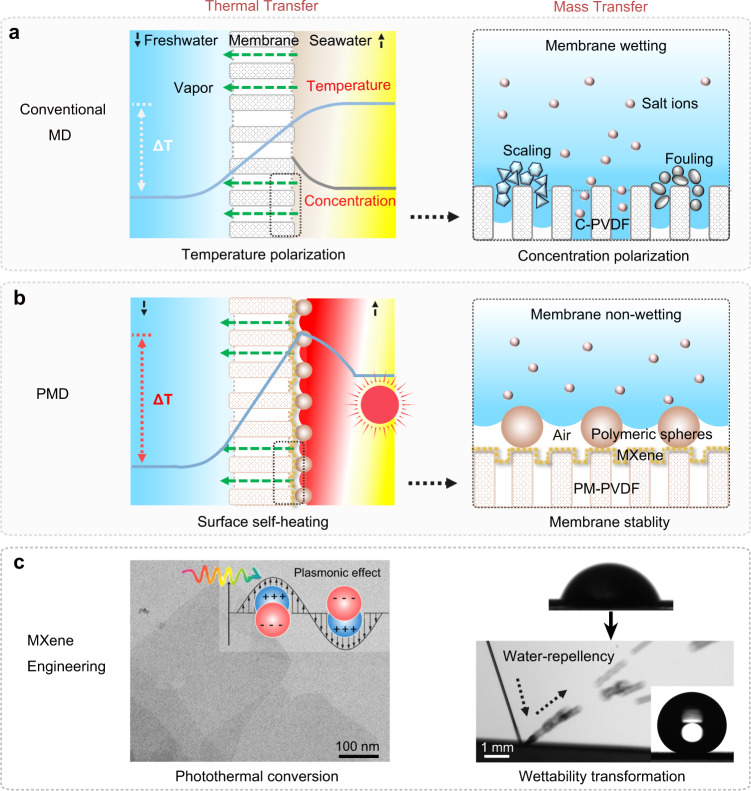
Design concept of the PM-PVDF membrane for PMD. **a** The conventional MD process with thermal inefficiency and membrane wetting issues using C-PVDF membrane due to the inherent temperature and concentration polarization effect. MD process involves hot seawater (yellow) and cold freshwater (blue) flow on the opposite sides of a porous hydrophobic membrane, which allows for vapor permeation established by cross-membrane temperature gradient ∆*T*, while rejects the liquid water and salt ions. The blue line represents the temperature polarization with a progressive decline of ∆*T* owing to inherent heat loss at the feedwater side as a result of continuous water-to-vapor transition. The yellow color shading represents temperature decline near the feed/membrane interface compared with the initial feed temperature (yellow). The gray line represents concentration polarization with a gradual increase of salts concentration close to the interface, which intensifies the propensity of wetting transition-induced membrane fouling and scaling. **b** Optimized PMD process with localized surface self-heating and superhydrophobic-dependent membrane stability endowed by PM-PVDF membrane. The PM-PVDF membrane enables the enhanced ∆*T* by photothermal effect and non-wetting performance by the superhydrophobic hierarchical MXene layer with polymeric nanospheres. The typical red color shading represents the temperature increase near the feed/membrane interface compared with the initial feed temperature (yellow), resulting from the localized surface heating through the photothermal effect. **c** MXene engineering that achieves photothermal conversion and wettability transformation from hydrophilicity to superhydrophobicity. Left: TEM image of MXene nanosheets and inset image shows the schematic plasmonic effect-enhanced photothermal conversion of MXene. Right: contact angles at the initial state and after engineering with strong water repellency.

Seawater desalination continues to be an integral part of the water portfolio to address the ever-increasing challenge of freshwater scarcity worldwide. Despite extensive efforts and notable progress, most existing technologies involve high energy expenditure and challenging brine management, especially when treating hypersaline solutions^1–5^. Recently, membrane distillation (MD), an emerging thermal-driven membrane-based process, has demonstrated significant advantages, such as high salt rejection, high water recovery from brine treatment through synergic integration with Reverse Osmosis (RO), and high compatibility with renewable energy sources^6–11^. In a typical direct contact membrane distillation (DCMD) process, hot saline water (feed) and cold purified water (permeate) flow on the opposite sides of a hydrophobic membrane, establishing a cross-membrane temperature gradient (∆*T*) that drives the water-to-vapor transition (Fig. 1a). Serving as both a medium for vapor transport and a barrier against direct liquid permeation, the hydrophobic membrane effectively allows vapor to transport from the feed side to permeate side and condense into freshwater, while rejecting liquid water and salt ions.

Fundamentally, an ideal MD process requires a high vapor permeate flux, which could be achieved through a constant cross-membrane temperature gradient (∆*T*) and good membrane durability. However, from the perspective of thermal transfer and mass transfer in a conventional MD process, two challenges largely hinder MD’s practical application. One daunting challenge is the difficulty in maintaining a constant ∆*T*. On the one hand, MD requires a continuous energy input to heat the bulk feedwater to create a constant ∆*T* and drive water-to-vapor transient. On the other hand, ∆*T* progressively declines due to the inherent heat loss at the feedwater side as a result of the continuous water-to-vapor transition. The temperature near the feed/membrane interface decreases, which is also known as temperature polarization (Fig. 1a, left), resulting in reduced driving force and overall energy inefficiency^12,13^. One effective solution to address this energy efficiency of MD is to maintain a high ∆*T* by developing a surface heating membrane with low conductivity^12–18^.

The other issue impeding the application of conventional MD lies in the difficulty in preventing wetting transition-induced membrane fouling and scaling during operation (Fig. 1a, right). In an ideal MD process, the hydrophobic membrane should be maintained free from pore blocking and wetting to achieve both efficient vapor permeation and high salt rejection. However, treating hypersaline solutions inevitably increases the concentration of salts (or any other constituents in the feed) close to the feed-membrane interface. This concentration polarization effect intensifies the membrane’s propensity to scaling, wetting, and fouling, leading to the decline in freshwater production rate and membrane failure^19–21^.

An efficient and sustainable strategy to address these challenges in conventional MD and harness the water-energy nexus could be the development of a photothermal MD (PMD) system^12–14,22–24^. The photothermal membrane’s continuous and localized surface self-heating, achieved by harvesting solar energy, could maintain the cross-membrane temperature gradient^25–28^. However, it remains a big challenge to construct an ideal PMD membrane that fundamentally possesses the highly desired combination of characteristics, including efficient photothermal effect and durable wetting resistance. Recent studies have reported the use of titanium carbide (Ti_3_C_2_T_x_, T_x_ = –F, –O, and –OH), an emerging two-dimensional (2D) MXene with interlayered 2D channels and plasmonic-enhanced photothermal capabilities, which enables great superiority for solar-driven freshwater production^29–33^. Nevertheless, the practical application of titanium carbide in MD is largely hindered by its intrinsic hydrophilicity, which inevitably causes membrane wetting and impedes desalination stability^34,35^.

Thus, this study attempts to present a feasible and effective strategy for designing a wetting-resistant MXene membrane to advance its potential application for treating hypersaline solutions. Specifically, we propose a photothermal Ti_3_C_2_T_x_ MXene-engineered polyvinylidene fluoride membrane (PM-PVDF) that imparts an efficient, localized photothermal effect and a strong water-repellency for sustainable PMD (Fig. 1b). Our design of interfacial engineering mainly makes use of MXene’s unique solar harvesting capability and high electrical conductivity to achieve both excellent photothermal conversion and wettability transformation (Fig. 1c). On the one hand, the photothermal MXene enables sustainable surface self-heating to circumvent heat loss at the feed side caused by continuous water-to-vapor transition, thereby maintaining a high and constant cross-membrane temperature gradient. On the other hand, the high electrical conductivity of MXene allows for the self-assembly of uniform hierarchical polymeric nanospheres via electrospraying on its surface, transforming its intrinsic hydrophilicity into superhydrophobicity without the need for any additional surface treatment. Leveraging on the functional interfacial engineering, our PM-PVDF membrane is endowed with highly desired energy-efficient and wetting resistance capability for hypersaline solution treatment, rendering a significant boost in freshwater production flux and stability.

## Results and discussion

### Design and fabrication of PM-PVDF membrane

**Fig. 2:**
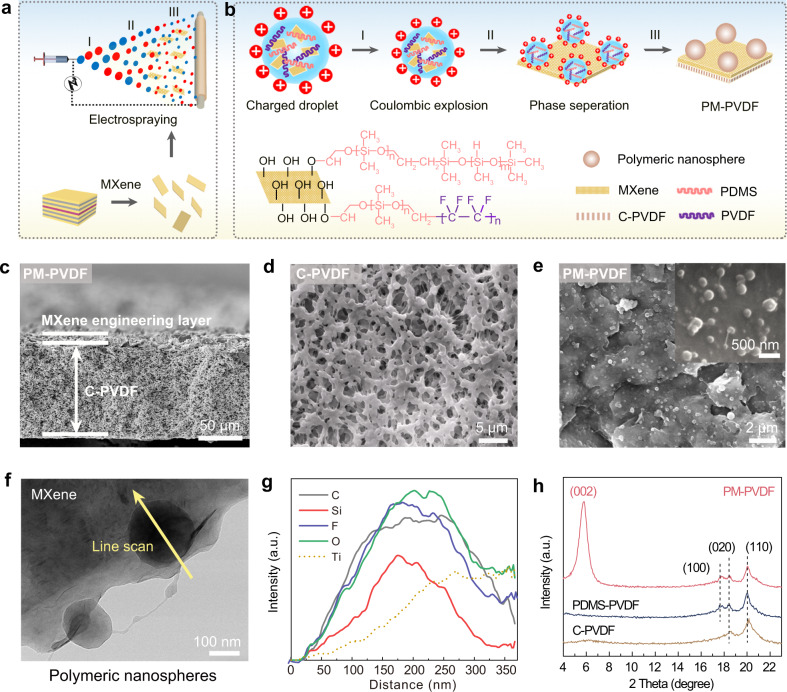
Fabrication and structural characterization of membranes. **a** A schematic illustration of the interfacial engineering to fabricate the PM-PVDF membrane. MXene was synthesized by etching-exfoliation method and then engineered onto C-PVDF membrane by a facile electrospraying process. **b** Electrically conductive MXene-directed self-assembly of polymeric nanospheres by electrospraying. The formation of polymeric nanospheres involves three steps including the formation of charged droplets, coulombic explosion, and phase separation. The high electrical conductivity and sufficient surface functional groups of MXene play a structure-directing for the assembly of polymeric nanospheres, which can only be generated when PDMS and highly molecular PVDF are both present. **c** Cross-sectional SEM image of the PM-PVDF. **d** SEM image of the C-PVDF and (**e**) PM-PVDF. **f** TEM image of 2D MXene nanosheets decorated with polymeric nanospheres. **g** Corresponding EDS line scan on the polymeric nanospheres with spherical elements distribution. **h** GIXRD patterns. Source data are provided as a Source Data file.

Our designed MXene-engineered membrane (PM-PVDF) is composed of two components, which are the interfacial polymeric nanosphere-assembled MXene-engineering layer and the base C-PVDF membrane layer. The PM-PVDF membrane was fabricated in two steps, as schematically shown in Fig. 2a. Ti_3_C_2_T_x_ MXene nanosheets were first synthesized using the HCl/LiF etching and exfoliation method, followed by a subsequent freeze-drying process (Supplementary Figs. 1–3). Then, the MXene engineering and its incorporation onto the C-PVDF membrane layer was simultaneously achieved by a facile electrospray process to form the PM-PVDF membrane. The electrospray engineering process mainly involves three steps: formation of charged droplets, coulombic explosion, and phase separation (Fig. 2b). We used polydimethylsiloxane (PDMS) and PVDF dispersed in the miscible solvent of THF and DMF as the polymer precursor for the Ti_3_C_2_T_x_ MXene electrospray solution. PDMS is hydrophobic and has low thermal conductivity with silane -Si-O- groups, which could generate sufficient nucleation sites via bonding with MXene’s surface functional groups (-OH, -O, and -F). The combination of PDMS and PVDF generates the preferred polymeric nanospheres that self-assemble under the structure-directing of MXene’s high electrical conductivity. Noted that these polymeric nanospheres can only be generated when PDMS and highly molecular PVDF are both present. Neither the PDMS nor PVDF alone could generate polymeric nanospheres (Supplementary Fig. 4) due to its low molecular weight and low surface energy.

The structure and morphology of the obtained PM-PVDF membrane was further characterized by SEM and TEM images. Figure 2c exhibits the cross-sectional structure of the PM-PVDF membrane, which consists of the C-PVDF bottom layer (Fig. 2d) and the MXene-engineering surface layer (Fig. 2e), respectively. The polymeric nanospheres that self-assembled on MXene (Fig. 2f) were verified by the TEM image and the corresponding elemental line-scan profile (Fig. 2g). The images display the uniform distribution of C, O, F, Si, and Ti elements in spherical shapes, related to polymeric nanospheres and Ti_3_C_2_T_x_ MXene. Notably, the MXene-directed polymeric spheres exhibit a much smaller average diameter (~200 nm) than those produced with PDMS and PVDF (P-PVDF) in the absence of MXene, which had micro-scale diameters of ~2 um (Supplementary Fig. 5).

The largely reduced size of the polymeric spheres verified the functionality of MXene based on high conductivity and abundant functional groups. The presence of MXene not only provides more nucleation sites, but also induces the Coulombic fission of electrostatic droplets during electrospray. Equation (1) below illustrates the factors that affect the size of the particles during electrospray^36^:

1 $${d}_{{{{{{\rm{p}}}}}}}={\varPhi }^{1/3}d \sim G\left(\varepsilon \right){\left(\tfrac{{{{{{{\rm{Q}}}}}}{{{{{\rm{\varepsilon }}}}}}{{{{{\rm{\varepsilon }}}}}}}_{0}}{{{{{{\rm{k}}}}}}}\right)}^{1/3}$$

where *d*_p_ and *d* is the diameters of the particle and droplet, respectively; Φ is the polymer volume fraction; *Q* is the liquid flow rate; *ɛ* and *ɛ*_0_ are the permittivity of liquid and vacuum, respectively; and *k* is the liquid conductivity. The formation of the MXene-engineering layer is further evidenced in the GIXRD pattern (Fig. 2h), which exhibits the characteristic (002) peak of MXene related to *d* spacing, as well as (100), (020), and (110) peaks that correspond to the coexistence of PDMS and PVDF. Also, the abundant molecular interactions shown in the FTIR spectra (Supplementary Fig. 6) and Raman spectra (Supplementary Fig. 7) further indicate the PM-PVDF membrane’s stable structure.

### Photothermal effect and thermal conductivity of the membrane

**Fig. 3:**
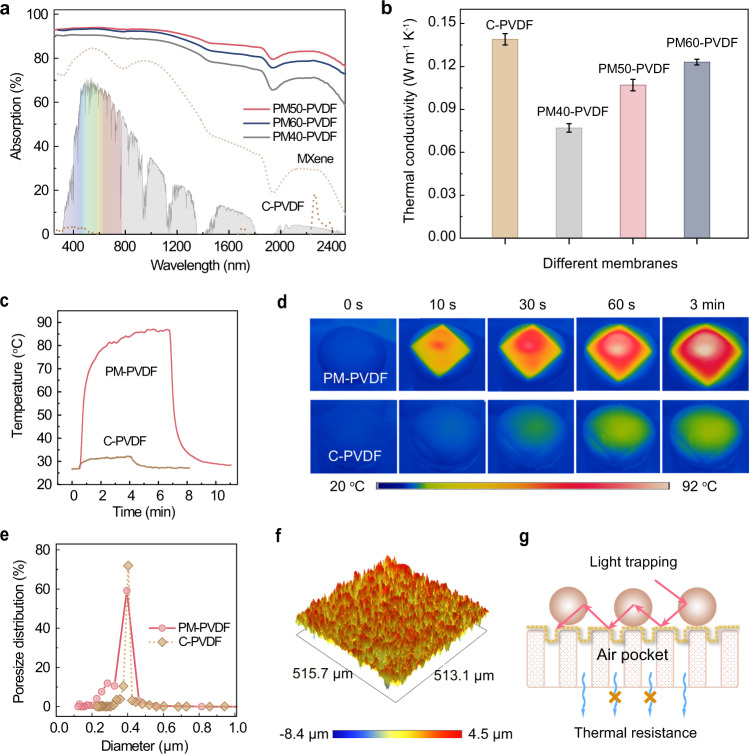
Photothermal effect and thermal conductivity of membranes. **a** UV-vis-NIR absorption spectra of the membranes. **b** The corresponding thermal conductivities. Error bars indicate the standard deviations estimated from three times measurements. **c** Surface temperature profiles as a function of time and (**d**) the corresponding IR thermal images. **e** Pore sizes distribution. **f** Typical optical surface profile of the PM-PVDF. **g** Schematic illustration of the PM-PVDF with favorable light trapping and thermal resistance. The hierarchical topology maximizes the sunlight harvesting by the light trapping effect, and meanwhile, the trapped air pockets in the membrane serve as thermal resistance to alleviate thermal conduction through the membrane and achieve efficient solar utilization. Source data are provided as a Source Data file.

A photothermal membrane that efficiently produces photothermal effects with low thermal conductivity enables localized surface heating on the feed side while alleviating unexpected heat conduction to the permeate side, which is desirable for maintaining a high cross-membrane temperature gradient. Figure 3a exhibits the UV-Vis-NIR absorption spectra of the fabricated membranes with optimized chemical components. The PM-PVDF membranes with varied MXene layer thicknesses demonstrated the distinct absorption of Ti_3_C_2_T_x_ MXene with two characteristic strong absorption peaks centered at 610 nm and 1148 nm, while the C-PVDF membrane showed negligible sunlight absorption capacity. Especially, the optimum PM50-PVDF membrane displayed stronger and broader solar absorption (~93.6%). Meanwhile, the PM-PVDF membranes demonstrated a lower thermal conductivity (0.08 ~ 0.12 W m^−1^ K^−1^) than the C-PVDF membrane (0.14 W m^−1^ K^−1^) (Fig. 3b), despite being incorporated with highly thermal conductive MXene. Consequently, the surface temperature of the PM50-PVDF membrane increased rapidly to 84.8 °C in 3 min when exposed to one-sun illumination (Fig. 3c), demonstrating its outstanding surface self-heating behavior. As further verified by the IR thermal images in Fig. 3d, the PM50-PVDF membrane delivered a temperature increment of 60.5 °C, which is almost 10 times than that of C-PVDF membrane (6.2 °C) under the same conditions. Conclusively, the photothermal layer imparted excellent localized self-heating while minimizing heat loss to alleviate the temperature polarization inherent in conventional DCMD.

We ascribe the combination of efficient photothermal effect and low thermal conduction to the multi-functional MXene engineering layer. The self-assembly of polymeric nanospheres on MXene rendered a topological texture with hierarchical structure and trapped air pockets, which was verified by the PM-PVDF membrane’s pore structure (Fig. 3e) and optical surface profiles (Fig. 3f). Moreover, the porosity before (C-PVDF) and after surface modification (PM-PVDF) were 69.2 ± 0.2% and 71.8 ± 0.3%, respectively. The slight increase in porosity after surface modification is due to the electrospray-induced hierarchical MXene layer with polymeric nanospheres. Besides, the increase in porosity with effective phonon scattering and the introduction of low thermal conductive PDMS synergistically gave rise to the relatively lower thermal conductivity of the PM-PVDF membrane. This combined functionality of the MXene layer can be schematically illustrated in Fig. 3g. From the perspective of solar absorption, the hierarchical topology is favorable for maximizing sunlight harvesting via nanoscale light trapping effect; from the perspective of thermal transfer, the hierarchical porous structure with trapped air pockets serves as thermal resistance to alleviate thermal conduction through the membrane and achieve efficient solar utilization.

### Superhydrophobic transformation with strong water repellency

**Fig. 4:**
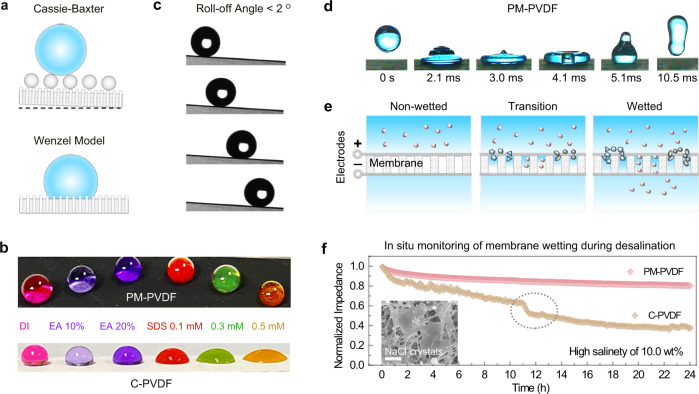
Water repellency and membrane wetting resistance. **a** A schematic illustration of a water droplet in the Cassie-Baxter state on the PM-PVDF and the Wenzel model state on the C-PVDF, respectively. **b** Photography of different solvent droplets on the C-PVDF and the PM-PVDF, respectively. From left to right: DI water, 10 and 20% ethanol (EA) in water, 0.1 mM, 0.3 mM and 0.5 mM sodium dodecyl sulfate (SDS) in water. **c** Low slide angle of the PM-PVDF. **d** Selected snapshots of a water droplet impinging on the superhydrophobic membrane surface. **e** Schematic illustration on wetting-induced membrane issues monitored by the evolution of membrane impedance during desalination. The wetting transition would induce a cross-membrane permeation of the salt ions from feedwater and a further accumulation of crystals inside of the membrane pores, which could be directly reflected by the decline of membrane’s impedance. **f** Normalized membrane impedances in situ monitored during hypersaline solution treatment. Scale bar: 5 μm. The dotted circle represents the transition stage of impedance accompanied by aggravating membrane wetting. Inset is SEM image of C-PVDF with the crystals scaling inside of the membrane pores. Source data are provided as a Source Data file.

The surface wettability of the membrane is another factor that affects desalination performance, which connects to wetting transition-induced membrane scaling and fouling. In this regard, the self-assembly of uniform hierarchical polymeric nanospheres on the MXene layer’s surface transforms MXene’s intrinsic hydrophilicity into superhydrophobicity without the need for any additional surface treatment. As aforementioned in Fig. 1c, after the wettability transformation by MXene engineering, the PM-PVDF membrane’s contact angle (CA) increased from ~70° to ~172°, which denotes a superhydrophobic surface in the Cassie-Baxter state (Fig. 4a). To the best of our acknowledge, the superhydrophobicity of our PM-PVDF membrane is the highest among reported MXene-engineered membranes. The superhydrophobic PM-PVDF membrane also ensured good repellence of low surface tension substances (Fig. 4b), maintaining spherical droplets when drops of ethanol and SDS were deposited. Conversely, both the pristine C-PVDF (CA = 125°) and MXene-absent P-PVDF (CA = 155°) membranes displayed hydrophobic performances in the Wenzel model state (Supplementary Fig. 8).

Furthermore, the PM-PVDF membrane exhibited a super low slide angle below 2° (Fig. 4c), which enabled water drops to roll off easily with negligible contact angle hysteresis, as further verified by the advancing and receding process (Supplementary Fig. 9). Another indication of strong water repellency is the water-bouncing phenomenon. The impinging droplet did not penetrate the superhydrophobic surface of the PM-PVDF membrane; instead, it rebound into the air in <10.05 ms and bounced more than 10 times before it finally rested on the surface (Fig. 4d). This superior water repellency is attributed to the low surface energy and roughness of the PM-PVDF membrane’s surface topology. In particular, the hierarchical polymeric nanosphere-MXene engineering interface generated a large fraction of porosity with air pockets that were trapped beneath water drops, which would construct a boundary slippage and stable shear-free water-air interface line during desalination^37,38^.

To characterize the PM-PVDF membrane’s wetting resistance during the treatment of high salinity solution (10 wt% NaCl), we in situ monitored the evolution of membrane impedance using an electrochemical workstation (Fig. 4e and Supplementary Fig. 10). Figure 4f shows the gradual decline of the control C-PVDF membrane’s impedance due to the wetting-induced cross-membrane permeation of the ions from feedwater, which further causes the accumulation of crystals inside of the membrane pores (inset of Fig. 4f and Supplementary Fig. 11). The transition stage of impedance is accompanied by the aggravating issues of membrane wetting, scaling, and fouling, which lead to rapid declines in freshwater production flux and salt rejection (Supplementary Fig. 12). Conversely, the superhydrophobic PM-PVDF membrane demonstrated stable membrane impedance. The PM-PVDF maintained consistent morphology and superhydrophobic performance (Supplementary Fig. 13), indicating its great potential for stable and efficient desalination of hypersaline solutions. This excellent wetting resistance of PM-PVDF membrane can be fundamentally explained by the breakthrough liquid entry pressure (Δ*P*) according to Young-Laplace equation^11^. The PM-PVDF membrane showed a higher Δ*P* of 2.05 ± 0.04 bar compared to that of the C-PVDF (1.18 ± 0.03 bar) due to the superhydrophobicity and smaller pore size endowed by the hierarchical MXene layer. This Δ*P* value is also consistent with the liquid entry pressure (LEP) measured using a porometer (POROLUX™ 1000) (Supplementary Table 1).

### Performance of hypersaline desalination by PMD

**Fig. 5:**
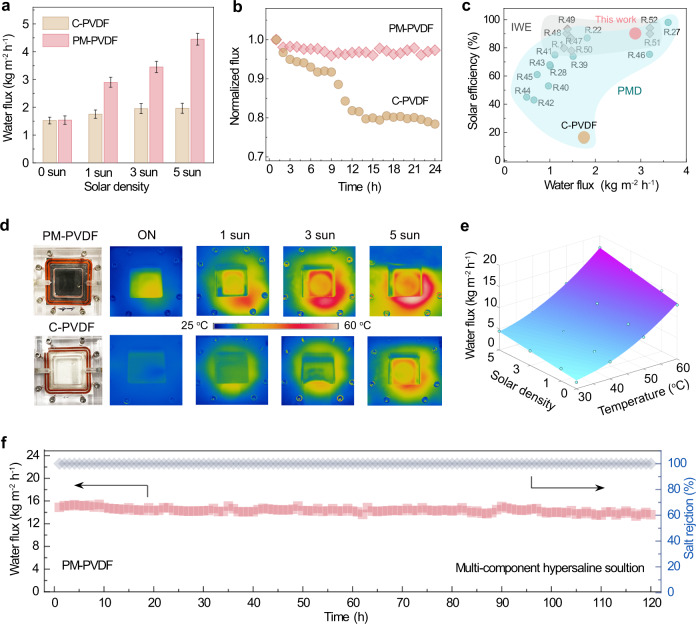
Demonstration on performance of hypersaline desalination by PMD. **a** Freshwater production flux of PM-PVDF and C-PVDF membrane under different sun densities. Feed, 30 ^o^C. **b** Stability evaluation of membrane in terms of normalized freshwater production flux. Error bars indicate the standard deviations estimated from three times measurements. **c** Summary of typical state-of-the-art solar desalination strategies operated in varied conditions. Solar efficiency is related to solar-to-vapor conversion or utilization around 1 sun. **d** Surface temperature of module monitored by IR thermal image during operation. **e** Phase diagram and simulated model for the performance of PM-PVDF membrane. **f** Long-term stability of the PM-PVDF during multi-component hypersaline solution treatment under 1 sun illumination. Feed, 60 ^o^C. Source data are provided as a Source Data file.

To further validate the PM-PVDF membrane’s desalination performance by PMD, we used a DCMD module with a light window (Supplementary Fig. 14) for the PMD treatment of a hypersaline solution. We first studied the freshwater production flux using a feed of 30 °C, which is close to the actual temperature of Hong Kong’s seawater in August. As shown in Fig. 5a, the PM-PVDF membrane with no solar illumination delivered an average freshwater production flux of ~1.55 kg m^−2^ h^−1^, which is comparable to that of the control C-PVDF membrane (1.53 kg m^−2^ h^−1^). However, when exposed to one sun illumination, the freshwater production flux of the PM-PVDF membrane increased to about 2.88 kg m^−2^ h^−1^, while the C-PVDF membrane displayed a negligible increase. A similar trend was also found under different operational conditions in terms of sunlight density. In addition, the apparent solar-to-vapor conversion efficiency (η) of the PM-PVDF was calculated to be about 89.0 %, which is much higher than that of the C-PVDF (~15.5%), indicating efficient photothermal conversion. Moreover, in contrast to the rapid decline in freshwater production flux experienced by the C-PVDF, the PM-PVDF demonstrated a much stable desalination performance (Fig. 5b), which is consistent with the evolution of membrane impedance shown in Fig. 4f. For comparison, we summarised part of the solar desalination performance of our PM-PVDF membrane and other existing studies in Fig. 5c. Overall, PM-PVDF membrane demonstrated many advantages when compared with state-of-the-art PMD membranes^22,27,28,39–46^ as well as solar-driven interfacial water evaporators (IWE)^1,47–52^ in terms of freshwater production flux and solar-to-vapor efficiency, especially for hypersaline solution treatment.

We further studied the effect of the photothermal localized surface heating at the membrane-water interface during the PMD operation. The surface temperatures of the PM-PVDF membrane when exposed to 1, 3, and 5 sun illumination at 30.0 °C feed are recorded in Fig. 5d. The PM-PVDF membrane reached high values of about 38.2, 44.8, and 58.6 °C, respectively, while the C-PVDF membrane showed almost no obvious temperature increment. Under the exemplified one sun illumination, the distillate outlet temperature of the PM-PVDF membrane demonstrated an increment of 1.71 °C, which is higher than those with no solar illumination and of the C-PVDF membrane (Supplementary Table 2). It can be expected that the increase in localized surface heating through the photothermal effect could further compensate, alleviate, and reverse the temperature polarization inherent in conventional MD. Furthermore, reversing the temperature polarization along the membrane would further enlarge cross-membrane temperature gradient to strengthen the driving force for vapor transfer.

We presumed that the operational conditions, including feed temperature and solar density, would largely affect the surface temperature to render different temperature gradients across the membrane. To reveal the relationship between these operational conditions and freshwater production flux (Fig. 5e), we further developed an empirical modeling based on the experimental results, which can be described as the following Eq. (2):

2 $$J(T,S)=\; 	8.82\times {10}^{-3}{T}^{2}-42.49\times {10}^{-2}T+18.21\times {10}^{-3}{TS}\\ 	+\,27.13\times {10}^{-2}S+6.64$$

where *T* and *S* represent the feed temperature (°C) and solar illumination density (sun), respectively. According to Eq. (2), the freshwater production flux of the PM-PVDF membrane in our system is a polynomial of the second degree to feed temperature and of the first degree to solar density.

Furthermore, we evaluated the PM-PVDF membrane’s performance for treating multi-component hypersaline solutions with sparingly soluble salts. Supplementary Fig. 15 shows that the PM-PVDF demonstrated stable membrane impedance and freshwater production flux when treating multi-component hypersaline solutions, indicating good wetting and scaling resistance. As further verified by the 3D OCT images and SEM images, the membrane’s morphology maintained its initial state with few salt crystals precipitating during desalination. On the contrary, the C-PVDF membrane suffered from serious membrane wetting and underwent deterioration due to salt precipitation, leading to a rapid decline in water production flux. In the end, to validate the durability of the PM-PVDF membrane, we carried out a long-term desalination test under one sun illumination using the same multi-component hypersaline feed at 60 °C. Figure 5f demonstrates the PM-PVDF membrane’s stable freshwater production flux at 14.38 kg m^−2^ h^−1^ and high salt rejection (≥99.9%) for 120 h, indicating its high stability and durability. These results further suggest that the superhydrophobic hierarchical MXene layer serves as an efficient armour for wetting resistance and provides a slippery, scaling-resistant interface for stable water/air contact^37,38^.

In conclusion, we developed a Ti_3_C_2_T_x_ MXene-engineered membrane (PM-PVDF) with efficient localized photothermal capabilities and superior water repellency for sustainable PMD. The efficient solar harvesting capability of MXene endowed a photothermally localized surface self-heating ability to the membrane to circumvent inherent heat loss at the feed side induced by the continuous water-to-vapor transition. Moreover, the high electrically conductive MXene played a structure-directing role by enabling the self-assembly of uniform hierarchical polymeric nanospheres on its surface *via* electrospraying, which transformed MXene’s intrinsic hydrophilicity into superhydrophobicity. Benefiting from the interfacial engineering, our PM-PVDF membrane was endowed with a constantly high cross-membrane temperature gradient as well as strong wetting resistance, which led to a significant boost in freshwater production rate and stability under one sun illumination. This work presents an effective route for realizing energy-efficient and hypersaline-stable PMD operations that could harness the water-energy nexus by developing a multifunctional superhydrophobic MXene-engineered membrane.

## Methods

### Fabrication of the PM-PVDF membrane

#### Preparation of Ti_3_C_2_T_x_ nanosheets

Briefly, we first synthesized Ti_3_C_2_T_x_ nanosheets based on an HCl/LiF etching and exfoliation method. Following the typical process, 0.5 g Ti_3_AlC_2_ (MAX, purity >98 wt%) was immersed in a HCl (9 M) and LiF (0.5 g) aqueous solution, and the resulting mixture was magnetically stirred at 35 °C for 24 h. The resulting Ti_3_C_2_T_x_ solution was washed and exfoliated in deionized water (DI), resulting in Ti_3_C_2_T_x_ MXene dispersion. After a further freeze-drying process, Ti_3_C_2_T_x_ MXene powder was obtained.

#### Fabrication of PM-PVDF membrane

An optimized electrospray solution was prepared consisting of 3.0 wt% PDMS (Sylgard 184), 2.0 wt% Polyvinylidene fluoride (PVDF, *M*_*w*_ =  530,000 g mol^–1^), and a miscible solvent of THF/DMF (w/w = 1:1), followed by stirring at 65 °C for 12 h. Subsequently, different loadings of MXene powder were added and uniformly dispersed. Afterward, the electrospray solutions were transferred to plastic syringes and fixed on the electrospray machine (ET-2535). A stable high voltage of 20 kV and feed rate of 1.0 ml h^−1^ was applied, with a commercial PVDF (C-PVDF) membrane as collector. The ambient temperature and RH were 25 ± 2 °C and 48  ±  3%, respectively. Membranes with different weight ratios of PDMS, PVDF, and MXene of PM-PVDF membrane (3:2:2, 3:2:2.5, and 3:2:3) were fabricated, which were denoted as PM40-PVDF, PM50-PVDF, PM60-PVDF, respectively. We also fabricated a series of PM-PVDF membranes with MXene layers of varied thicknesses ranging from 5 ± 1.5 μm, 10 ± 1.5 μm, 15 ± 1.3 μm, to 25 ± 2 μm (Supplementary Fig. 16). Based on the membranes’ solar absorption, MXene consumption, and heat and mass transfer, we determined that the optimal thickness for the PM-PVDF membrane is about 110 ± 1.5  μm, composed of a 10 μm surface MXene layer and a 100 μm based layer (Supplementary Fig. 17). We used this PM-PVDF membrane, referred to as the optimum PM50-PVDF membrane, for tests unless specifically noted. An electrospray membrane without MXene was also prepared for comparison, denoted as the P-PVDF membrane. The C-PVDF membrane (0.45 μm, HVHP) from Millipore Company was used as a control membrane in the assessment of PMD performance.

### Characterization

The morphology and structures of the samples were characterized by transmission electron microscopy (TEM; JEOL 2011F) and field-emission scanning electron microscopy (FESEM; FEI Quanta 450). Grazing incidence X-ray diffraction (GIXRD; Ultima IV) was used to determine the crystalline structure of the membrane samples. Water contact angle measurements were performed on a drop-shape analyser (FM 4000, Krüss). Raman spectrometry was conducted on a Renishaw-200 visual Raman microscope using a 633-nm laser beam. Attenuated Total Reflection Fourier Transform Infrared spectroscopy (ATR-FTIR; 6700, Thermo Fisher) was used to examine the surface functional groups of the membranes. Absorption spectra were measured in the range of 250–2500 nm on an ultraviolet-visible spectrometer (UV–Vis 3600, Shimadzu) equipped with an integrating sphere. The thermal conductivities of the membranes were measured by a Mathis TCi thermal conductivity analyser (C-Therm). The membrane surface topology and roughness were characterized by a surface optical profiler (Wyko NT9300, Vecco). Pore size distribution was measured using a porometer (POROLUX™ 1000, Belgium). Solar-driven experiments were carried out using a Xe short-arc lamp (CEL-HXF300) as the illuminate, which was equipped with an optical filter for the standard AM 1.5 G spectrum. The thermal images and surface temperature distribution were recorded by an IR thermal camera (Optris PI 640). The porosities of the membranes were measured by the gravimetric procedure. Typically, the weight of the membrane sample (size: 3 cm × 3 cm) was measured under dry condition and fully 1-butanol wetted (W_1_) conditions, respectively. Then, the porosity of the membrane *ɛ* was determined using Eq. (3):

3 $$\varepsilon =\frac{\frac{{W}_{1}-{W}_{2}}{{D}_{1}}}{\frac{{W}_{1}-{W}_{2}}{{D}_{1}}\,+\,\frac{{W}_{2}}{{D}_{2}}}$$

where *W*_1_ and *W*_2_ represent the wet and dry weights (g) of the membrane, respectively, and *D*_1_ and *D*_2_ denote the densities of 1-butanol and polymer (g m^−3^), respectively.

The breakthrough pressures (Δ*P*) or the liquid entry pressure (LEP) of the membranes in water were calculated according to Young-Laplace equation:

4 $$\triangle P=-\frac{2\,\gamma \varTheta \,{{\cos }}{{\theta }}}{r}$$

where *γ* is the interfacial tension, *Θ* is a geometric factor related to the pore structure (equal to 1 for cylindrical pores), *θ* is the liquid–intrinsic contact angle, and *r* is the pore size of the membrane.

### PMD performance measurement

Membrane performance was evaluated using a custom-made lab-scale DCMD apparatus. The flat sheet membrane apparatus had a membrane area of 36 cm^2^ (6.0 cm × 6.0 cm). Hot feed and cold distillate streams were circulated using two variable gear pumps driver (Cole-parmer, Model 75211-15) and their temperatures were controlled using a heater (IKA, C-MAGHS7) and a chiller (Lab Companion, PW-0525G), respectively. Membrane performance at different feed temperatures (~30 °C, 40 °C, 50 °C, and 60 °C) was evaluated at a constant distillate temperature of 20 °C controlled by a chiller. The flow rate of the feed and the permeate side was about 100 ml/min, which generates a modest shear stress with a pressure below 0.02 bar. The conductivity of the feed and permeate was monitored using a conductivity meter (EUTECH, PC 2700), and membrane flux was determined by monitoring the weight increase on the distillate side using an electric balance (KERN PLE, 4200-2N). We used the thermoelectric couple (CEM, DT-3891G) to record the outlet temperatures of the feed and distillate side and a pressure probe (MEACON, MIK-Y190) to measure the pressure in the setup. The artificial multi-component feed solution consisted of sparingly soluble salts CaCO_3_ and CaSO_4_ at saturation concentration and a 10.0 wt% NaCl solution.

The membrane impedance during MD operation was in situ measured based on electrochemical impedance spectroscopy (EIS) using Autolab PGSTAT302N with FRAM32M module (Metrohm), and a carbon cloth was used as the electrodes to collect the electrochemical signals. The progress of membrane scaling was in situ evaluated using 3D optical coherence tomography (3D OCT, GANYMEDE-II OCT system). The salt rejection rate R (%) can be approximately calculated by:

5 $$R=\left(1-\frac{{C}_{p}}{{C}_{f}}\right)\times 100 \%$$

where *C*_*f*_ (mg L^−1^) and *C*_*p*_ (mg L^−1^) are the salt concentration of the feed side and the distillate side, respectively. Membrane flux (*J*, kg m^−2^ h^−1^) was calculated using the following equation to evaluate the freshwater production flux:

6 $$J=\frac{\triangle {W}_{p}}{\triangle t\,{A}_{m}}$$

where ∆*W*_*p*_ (kg) is the weight change in the distillate during period ∆*t* (h) and *A*_*m*_ (m^2^) is the effective membrane area. In addition, apparent solar-to-vapor conversion efficiency (*η*) was used to evaluate the water distillation performance, which can be defined as:

7 $$\eta =\frac{({J-J}^{{{\prime} }}){h}_{{LV}}}{\,{q}_{i}}$$

where *J* and *J′* represent the freshwater production flux (kg m^−2^ h^−1^) with and without sunlight illumination, respectively, *h*_LV_ denotes the ideal liquid-vapor phase change enthalpy (kJ kg^−1^), and q_i_ is the density of solar illumination (kJ m^−2^ h^−1^).

## Supplementary information

Supplementary Information

## Data Availability

The data that support the findings of this study are available within the article and Supplementary Information. Source data are provided with this paper.

## Source data

Source Data file
