# Supplementary material for: Transforming Ti3C2Tx MXene’s intrinsic hydrophilicity into superhydrophobicity for efficient photothermal membrane desalination
Source: Nat Commun. 2022 Jun 8;13:3315. doi: 10.1038/s41467-022-31028-6 (PMC9177613; doi:10.1038/s41467-022-31028-6)
Supplement: Supplementary file 1 — Supplementary Information [file 41467_2022_31028_MOESM1_ESM.pdf]

Supplementary Information

Transforming $Ti_3C_2T_x$ MXene's intrinsic hydrophilicity into superhydrophobicity for efficient photothermal membrane desalination

A. Kyoungjin An et al.

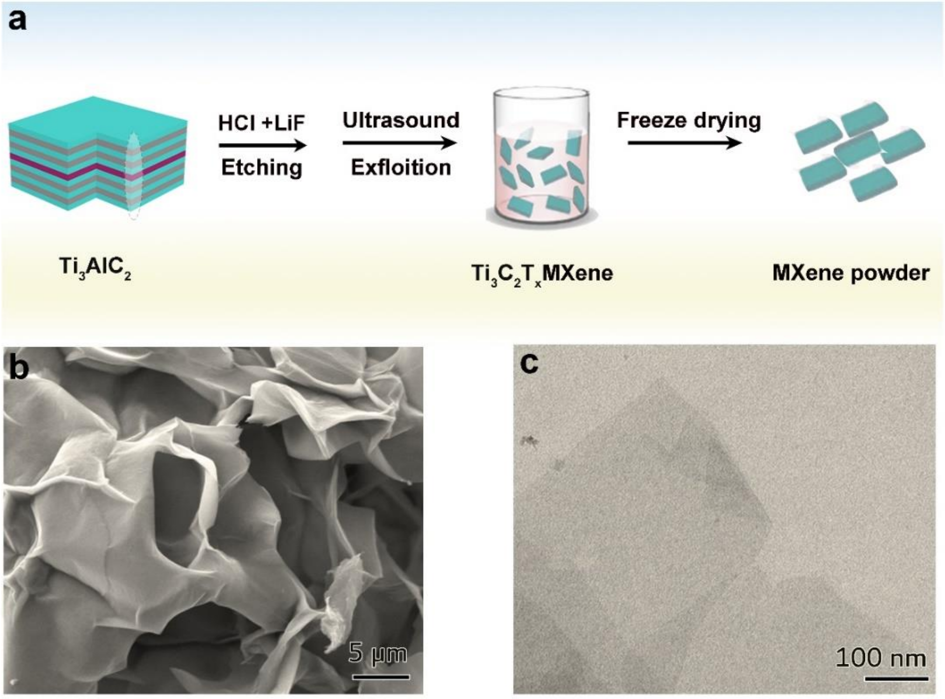

Supplementary Fig. 1 (a) A schematic illustration of the synthesis process of MXene powder. (b) SEM image and (c) TEM image of MXene.

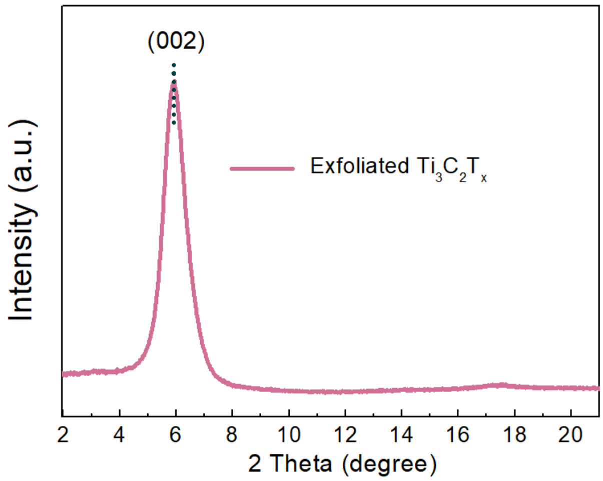

Supplementary Fig. 2 The XRD pattern of as-prepared $Ti_3C_2T_x$ MXene demonstrating the characteristic peaks of MXene at (002). Source data are provided as a Source Data file.

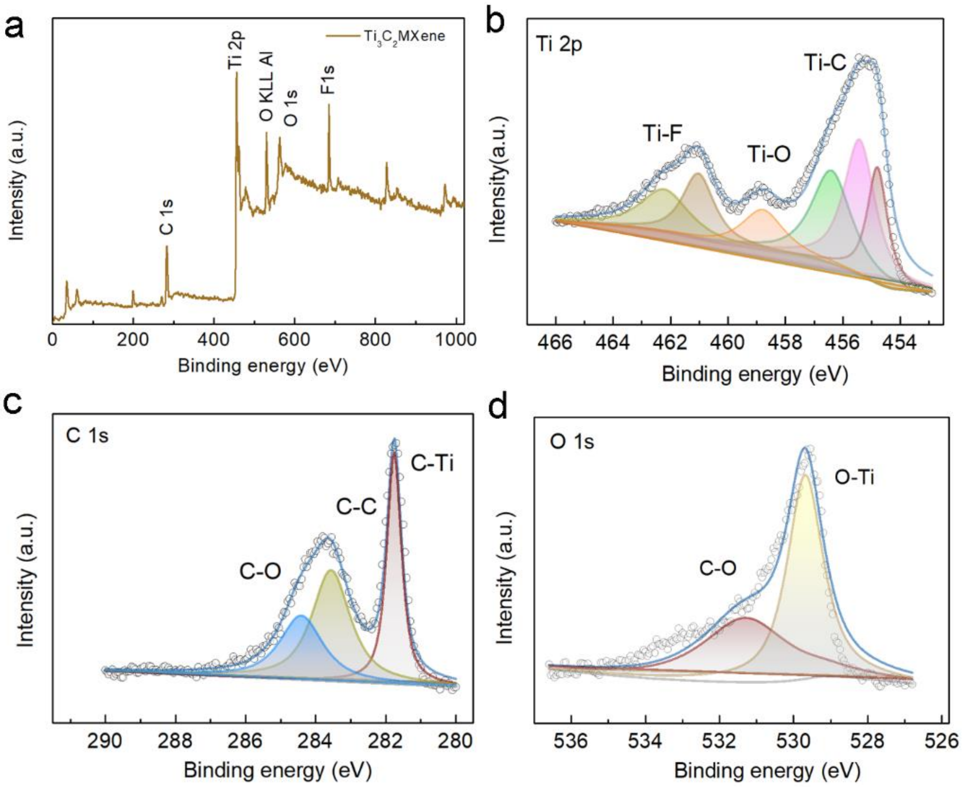

Supplementary Fig. 3 (a) XPS spectra of $Ti_3C_2T_x$ MXene. (b-d) High-resolution XPS spectra of Ti 2p, C1s, and O1s. Source data are provided as a Source Data file.

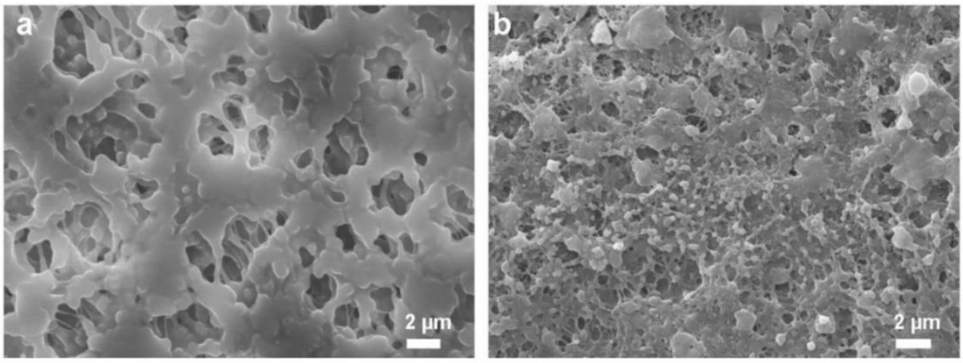

Supplementary Fig. 4 SEM images of the C-PVDF membrane with solely (a) PDMS electrospray coating and (b) PVDF electrospray coating on the surface. It is difficult to achieve polymeric spheres based on a sole polymer by electrospray.

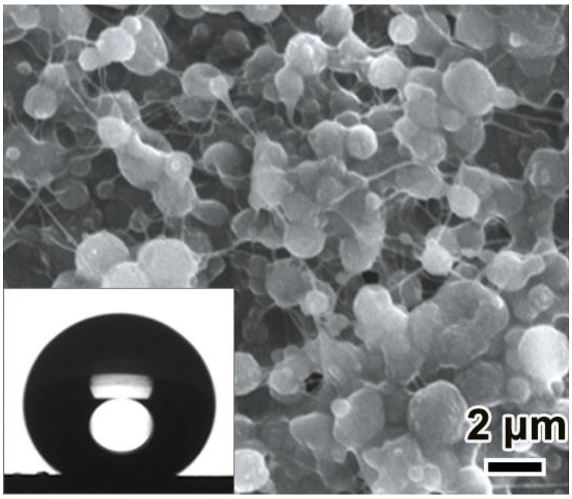

Supplementary Fig. 5 SEM image of the electrosprayed polymeric microspheres on the C-PVDF membrane (P-PVDF) in the absence of MXene with an average size of 2 μm. Inset is the contact angle of the P-PVDF membrane.

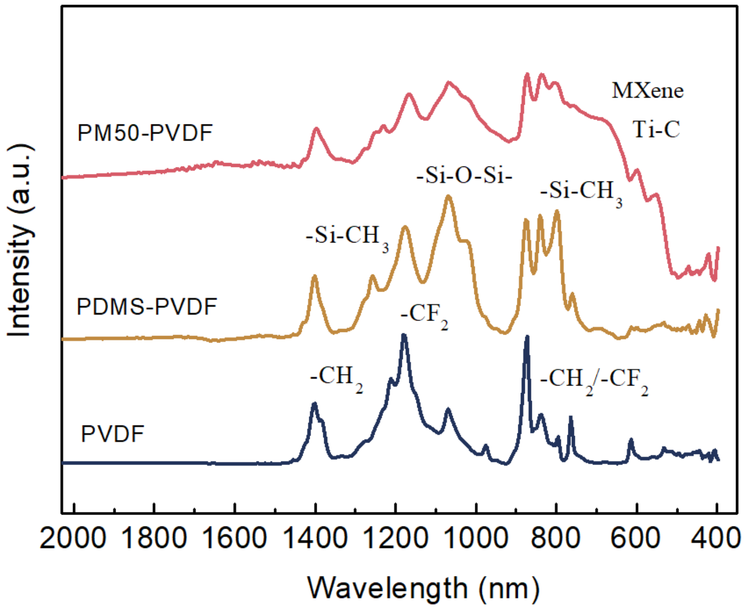

Supplementary Fig. 6 ATR-FTIR spectra of the C-PVDF, PDMS-PVDF, and PM-PVDF membranes. The obtained PM-PVDF membrane demonstrated the characteristic peaks corresponding to the functional groups of PVDF and PDMS. Source data are provided as a Source Data file.

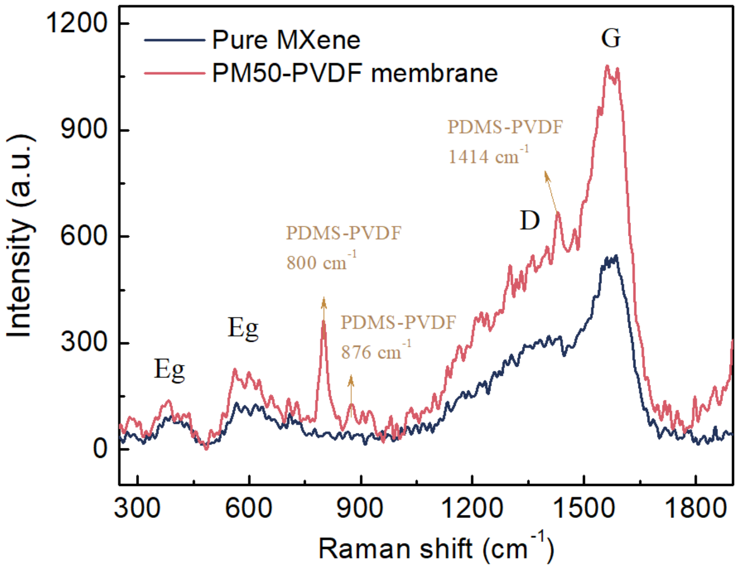

Supplementary Fig. 7 Raman spectra of the pure MXene membrane and the obtained PM50-PVDF membrane. The characteristic peaks of the Eg group correspond to the vibrations of $Ti_3C_2T_x$. The characteristic peaks at 800 cm $^{-1}$, 876 cm $^{-1}$ and 1414 cm $^{-1}$ corresponding to the presence of PDMS-PVDF in the composite membrane. Source data are provided as a Source Data file.

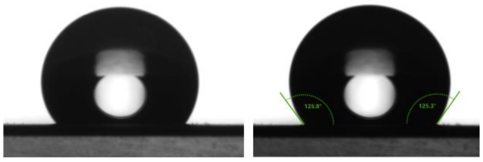

Supplementary Fig. 8 Contact angle of the C-PVDF membrane with 125 ^{o}.

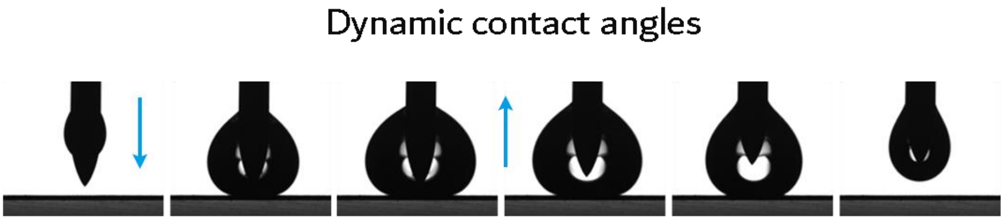

Supplementary Fig. 9 Dynamic contact angles during the advancing and receding process of the PM-PVDF membrane.

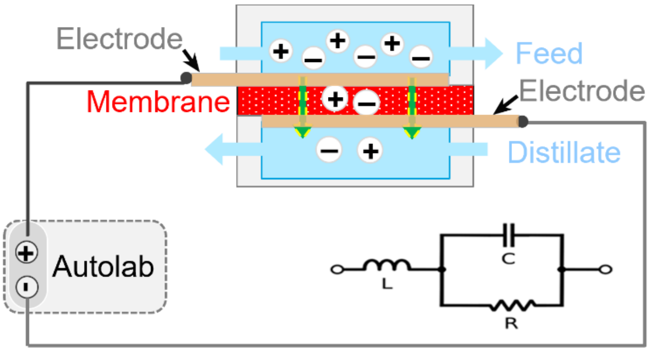

Supplementary Fig. 10 The schematic diagram of the MD setup with a Potentiostat (Autolab) to detect the changes in impedance for an *in-situ* monitoring of membrane wetting. Carbon cloth electrodes were inserted to both the feed and permeate sides of the membrane. The inset is the equivalent circuit diagram.

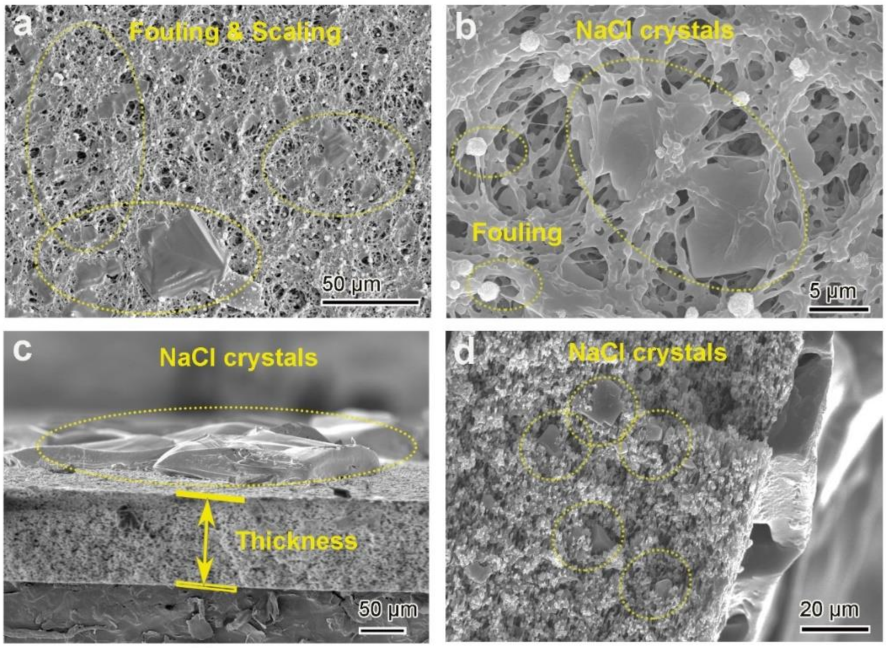

Supplementary Fig. 11 (a, b) SEM images and (c, d) cross-sectional SEM images of the C-PVDF membrane due to membrane scaling and fouling issues. As shown, abundant and various salt crystals have formed within or on the surface of the C-PVDF membrane due to membrane wetting and scaling issues.

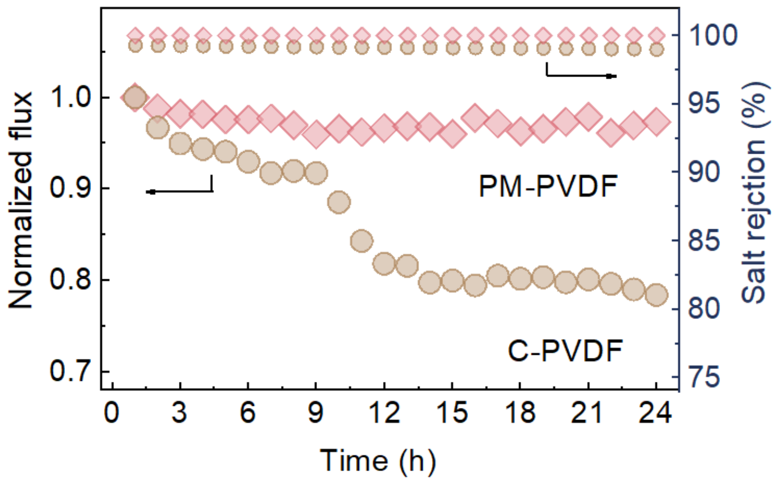

Supplementary Fig. 12 Comparison of normalized water production flux and salt rejection of the C-PVDF and PM-PVDF membranes while treating high salinity brine of 10 wt% NaCl. Source data are provided as a Source Data file.

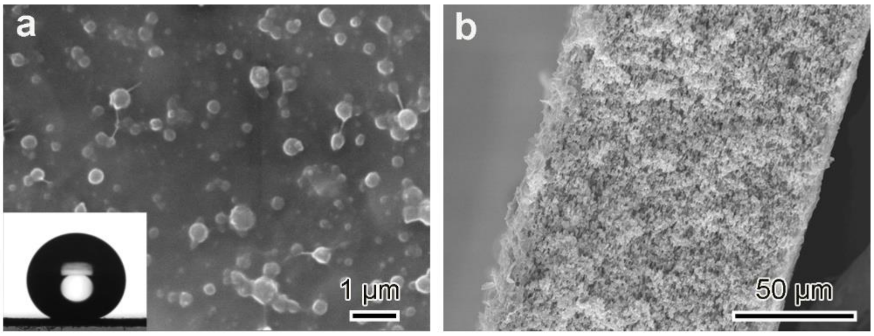

Supplementary Fig. 13 (a) SEM image and (b) cross-sectional image of the PM-PVDF membrane after desalination. Inset of (a) is the contact angle.

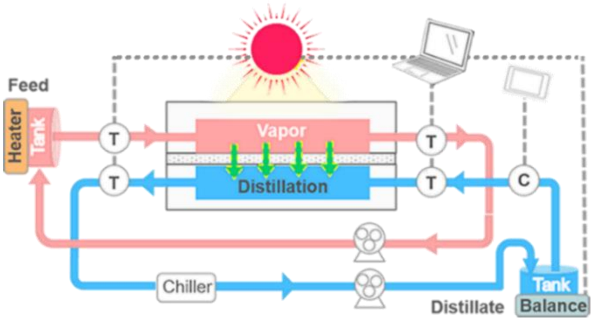

Supplementary Fig. 14 A schematic illustration of the home-made PMD set-up.

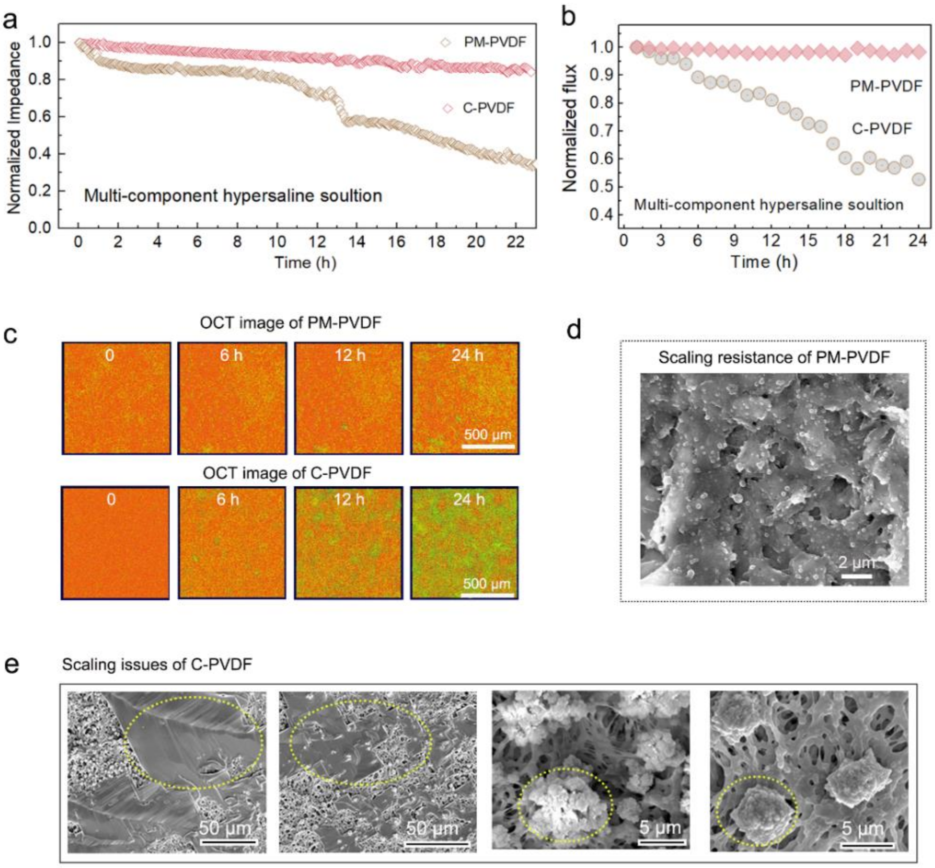

Supplementary Fig. 15 (a) Normalized membrane impedance of the C-PVDF and PM-PVDF membranes *in-situ* monitored during the treatment of a multi-component hypersaline solution. Wetting-induced cross-membrane permeation of the ions from feed water results in a decline in membrane impedance. (b) The corresponding normalized water production flux. (c) Scaling evolution of the membranes *in-situ* monitored by 3D OCT, where the green color corresponds to salt precipitation. (d) SEM images of the PM-PVDF membrane and (e) the C-PVDF membrane after desalination. Feed, 60°C. Source data are provided as a Source Data file.

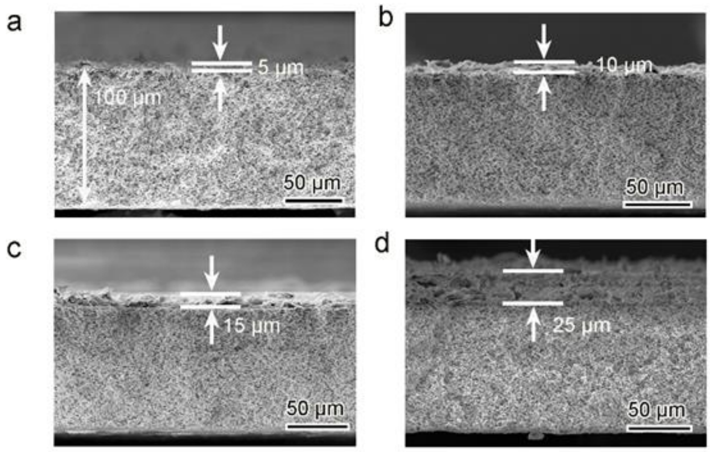

Supplementary Fig. 16 Cross-sectional images of PM-PVDF membranes with MXene layers of varied thicknesses: (a) 5 µm, (b) 10 µm, (c) 15µm, and (d) 25 µm. The thickness of the base C-PVDF layer is 100 µm.

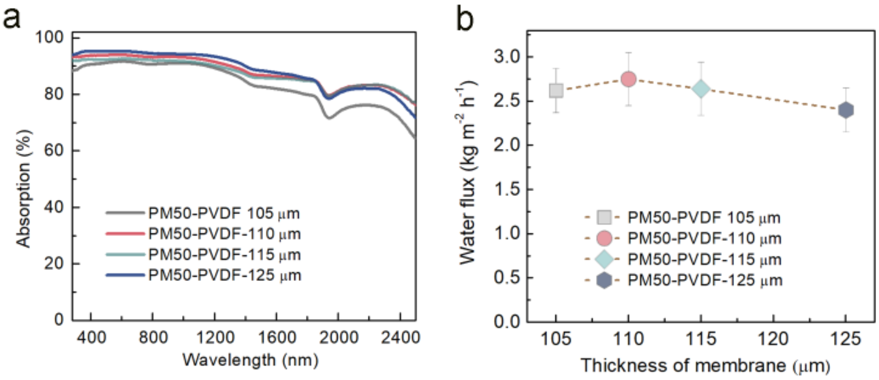

Supplementary Fig. 17 (a) The corresponding solar absorption and (b) freshwater production flux of the PM-PVDF membranes with different thicknesses under one sun illumination, feed 30 ºC. Error bars indicate the standard deviations estimated from three times measurements. Source data are provided as a Source Data file.

Supplementary Table 1 The measured LEP values of the C-PVDF and PM-PVDF membranes.

Membranes	Measured LEP（bar）
C-PVDF	1.26 ± 0.02
PM-PVDF	2.10 ± 0.05

Supplementary Table 2 Comparison of the feed and outlet temperatures of the C-PVDF and PM-PVDF membranes with no sun and one sun illumination, respectively.

Membranes	Solar density (sun)	$T_{f\text{-}in}$ (ºC)	$T_{f\text{-}out}$ (ºC)	$T_{d\text{-}in}$ (ºC)	$T_{d\text{-}out}$ (ºC)	$\Delta T_{distillate}$
C-PVDF	0	30.41	28.38	19.52	20.94	1.42
PM-PVDF	0	30.39	28.37	19.55	20.95	1.40
C-PVDF	1	30.51	28.55	19.66	21.13	1.47
PM-PVDF	1	30.52	28.63	19.63	21.34	1.71
